# Liquid Biopsy as Surrogate for Tissue for Molecular Profiling in Pancreatic Cancer: A Meta-Analysis Towards Precision Medicine

**DOI:** 10.3390/cancers11081152

**Published:** 2019-08-10

**Authors:** Claudio Luchini, Nicola Veronese, Alessia Nottegar, Vera Cappelletti, Maria G. Daidone, Lee Smith, Christopher Parris, Lodewijk A. A. Brosens, Maria G. Caruso, Liang Cheng, Christopher L. Wolfgang, Laura D. Wood, Michele Milella, Roberto Salvia, Aldo Scarpa

**Affiliations:** 1 Department of Diagnostics and Public Health, Section of Pathology, University of Verona, 37134 Verona, Italy; 2 National Institute of Gastroenterology-Research Hospital, IRCCS “S. de Bellis”, Castellana Grotte, 70013 Bari, Italy; 3 Department of Surgery, Section of Pathology, San Bortolo Hospital, 36100 Vicenza, Italy; 4 Applied Research and Technological Development Department, Fondazione IRCCS Istituto Nazionale dei Tumori di Milano, 20133 Milano, Italy; 5 Faculty of Science and Engineering, Anglia Ruskin University, Cambridge CB1 1PT, UK; 6 Department of Pathology, University Medical Center Utrecht, Utrecht University, 3584CX Utrecht, The Netherlands; 7 Department of Pathology, Radboud Institute for Molecular Life Sciences, Radboud University Medical Center, 6526GA Nijmegen, The Netherlands; 8 Department of Pathology and Laboratory Medicine, Indiana University School of Medicine, Indianapolis, IN 46202, USA; 9 Department of Surgery, Sol Goldman Pancreatic Cancer Research Center, The Johns Hopkins University School of Medicine, Baltimore, MD 21211, USA; 10 Department of Pathology, Sol Goldman Pancreatic Cancer Research Center, The Johns Hopkins University School of Medicine, Baltimore, MD 21211, USA; 11 Department of Oncology, Sol Goldman Pancreatic Cancer Research Center, The Johns Hopkins University School of Medicine, Baltimore, MD 21211, USA; 12 Department of Medicine, Section of Medical Oncology, University and Hospital Trust of Verona, 37134 Verona, Italy; 13 Department of General and Visceral Surgery, The Pancreas Institute, University and Hospital Trust of Verona, 37134 Verona, Italy; 14 ARC-Net Research Center, University of Verona, 37134 Verona, Italy

**Keywords:** liquid biopsy, cfDNA, pancreatic cancer, precision medicine, circulating tumor cells (CTC)

## Abstract

Liquid biopsy (LB) is a non-invasive approach representing a promising tool for new precision medicine strategies for cancer treatment. However, a comprehensive analysis of its reliability for pancreatic cancer (PC) is lacking. To this aim, we performed the first meta-analysis on this topic. We calculated the pooled sensitivity, specificity, positive (LR+) and negative (LR−) likelihood ratio, and diagnostic odds ratio (DOR). A summary receiver operating characteristic curve (SROC) and area under curve (AUC) were used to evaluate the overall accuracy. We finally assessed the concordance rate of all mutations detected by multi-genes panels. Fourteen eligible studies involving 369 patients were included. The overall pooled sensitivity and specificity were 0.70 and 0.86, respectively. The LR+ was 3.85, the LR- was 0.34 and DOR was 15.84. The SROC curve with an AUC of 0.88 indicated a relatively high accuracy of LB for molecular characterization of PC. The concordance rate of all mutations detected by multi-genes panels was 31.9%. LB can serve as surrogate for tissue in the molecular profiling of PC, because of its relatively high sensitivity, specificity and accuracy. It represents a unique opportunity to be further explored towards its introduction in clinical practice and for developing new precision medicine approaches against PC.

## 1. Introduction

Pancreatic cancer is a lethal malignancy with an increasing incidence, and it is projected to become the second most common cause of cancer-related death in the world at the end of the next decade [1,2,3]. The most common type of pancreatic cancer is represented by ductal adenocarcinoma (PDAC), which is responsible for more than 95% of deaths from pancreatic cancer [3,4].

At the time of diagnosis, the vast majority of patients with PDAC present with locally advanced or metastatic disease and are thus not amenable to surgical resection with a curative purpose [3]. For such patients, the priority is to receive an undebatable diagnosis of cancer including useful information for addressing all potential therapeutic approaches. However, obtaining tissue for the diagnosis in this setting may be difficult and requires invasive procedures. The gold standard is (endoscopic-) ultrasound guided fine-needle aspiration (US-FNA), which provides a limited number of cells for cytological analysis, not always allowing complete molecular profiling [5,6]. Moreover, the abundance of tumor stroma in PDAC impairs the negative predictive value of US-FNA because of sampling error, sometimes requiring a repeat of the procedure with further risks for PDAC patients [6,7].

For all these reasons, there is an urgent need for new techniques or biomarkers to aid in diagnosis, staging and clinical-therapeutic decisions, overcoming contraindications associated with invasive procedures. To date, the unique widely available blood-based test for patients with PDAC is the determination of the carbohydrate antigen 19-9 (CA19-9); however, because of its limited specificity, it is used only as generic support for diagnosis and for surveillance of recurrence [8].

Notably, recent studies have explored the possibilities of obtaining from the blood useful information for cancer treatment analyzing circulating tumor cells (CTCs), circulating-free DNA (cfDNA) or RNA, exosomes and secretomes, all parameters that are comprised under the general definition of liquid biopsy (LB) [9,10,11]. This kind of non-invasive approach represents a promising tool for new precision medicine strategies, since LB may be used also for molecular profiling [12,13,14,15].

Although some studies have explored and highlighted the usefulness of LB for genetic analysis of patients with PDAC [16,17,18,19,20], a comprehensive analysis of its reliability for this tumor type is lacking. With this first systematic review and meta-analysis on this topic, we aimed to highlight the reliability of LB in assessing the molecular profile of PDAC in comparison with the mutational analysis on tissue specimen. In addition, a specific focus on *KRAS* mutational status is provided, as this gene is the most frequently mutated driver gene in PDAC.

## 2. Results

### 2.1. Search Results and Descriptive Findings

Among 387 potential eligible studies, 60 full text articles were retrieved. Of them, 14 studies were eligible for this meta-analysis (Figure S1, Table 1) [21,22,23,24,25,26,27,28,29,30,31,32,33,34].

As reported in Table 1, the 14 studies eligible included 369 participants with pancreatic cancer, with a higher prevalence of stage I–II (57%) [21,22,23,24,25,26,27,28,29,30,31,32,33,34]. Eleven studies made the LB assessment at the same time as the tissue sample [21,22,23,24,25,27,28,30,31,33,34], and the remaining three at different time points [26,29,32]. The choice of tissue type for mutational analysis has been clearly specified in 11 studies: four studies used PDAC tissue from US-FNA [25,28,29,34], three from surgical resection [23,24,32] and four used PDAC tissue from US-FNA in case of metastatic or locally advanced PDAC and surgical resected tissue in case of early stage-PDAC [21,22,27,30]. The choice of the material as LB was represented in the vast majority of studies (12/14, 86%) by circulating-free DNA (cfDNA) from blood [21,22,23,24,25,27,28,29,30,32,33,34], whereas only two studies explored the use of circulating tumor cells (CTCs) for the same aim [26,31]. Eleven studies used polymerase chain reaction (PCR)-based techniques for the mutational analysis of *KRAS* status [21,22,23,24,25,26,27,28,30,31,33], whereas three studies performed next-generation sequencing (NGS) for a wider molecular analysis [29,32,34]. Overall, we identified 174 TP, 12 FP, 73 TN, 110 FN.

### 2.2. Quality of the Studies Included

As shown in Table S1, the risk of bias in the studies included is low, having only two studies reporting possible high risk of bias for flow and timing. However, it is unlikely that this affects our results since our stringent criteria for the selection of the studies ensure the high quality of the studies and of the specific procedures that have been performed.

### 2.3. Diagnostic Accuracy of the Liquid Biopsy

**Figure 1:**
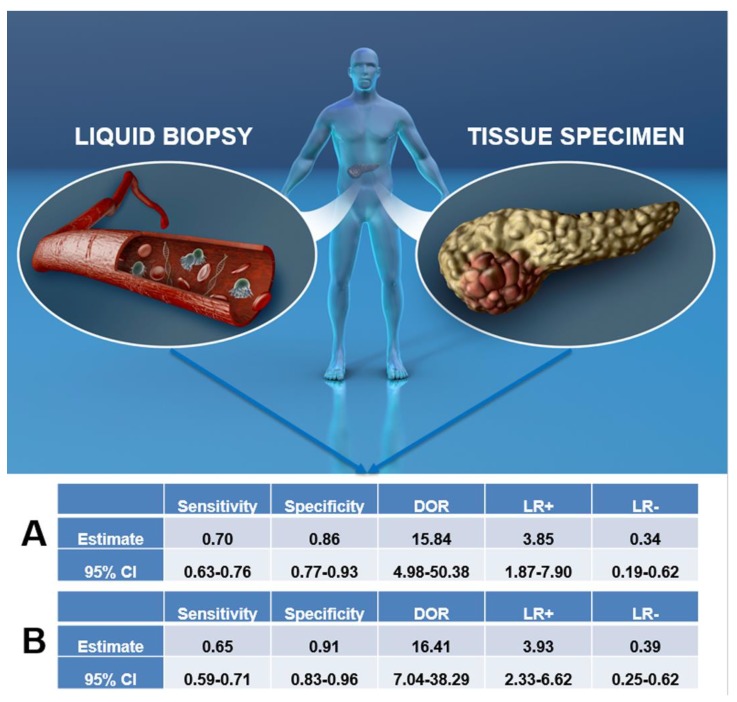
This figure represents the core of this meta-analysis, in which we tested the reliability of liquid biopsy to serve as surrogate for tissue for molecular profiling of pancreatic cancer. The upper panel is a schematic representation of the differences between the materials used for liquid biopsy (above all from the blood, on the left) and for tissue specimen analysis (surgical resections or cytology, on the right). The section indicated with letter A shows the overall summary of diagnostic accuracy parameters for liquid biopsy compared to tissue specimen with this meta-analysis, whereas the section with letter B shows the same parameters, but obtained analyzing data regarding *KRAS* only. Abbreviations: CI: confidence interval; DOR: diagnostic odds ratio; LR+: positive likelihood ratio, LR−: negative likelihood ratio.

**Figure 2:**
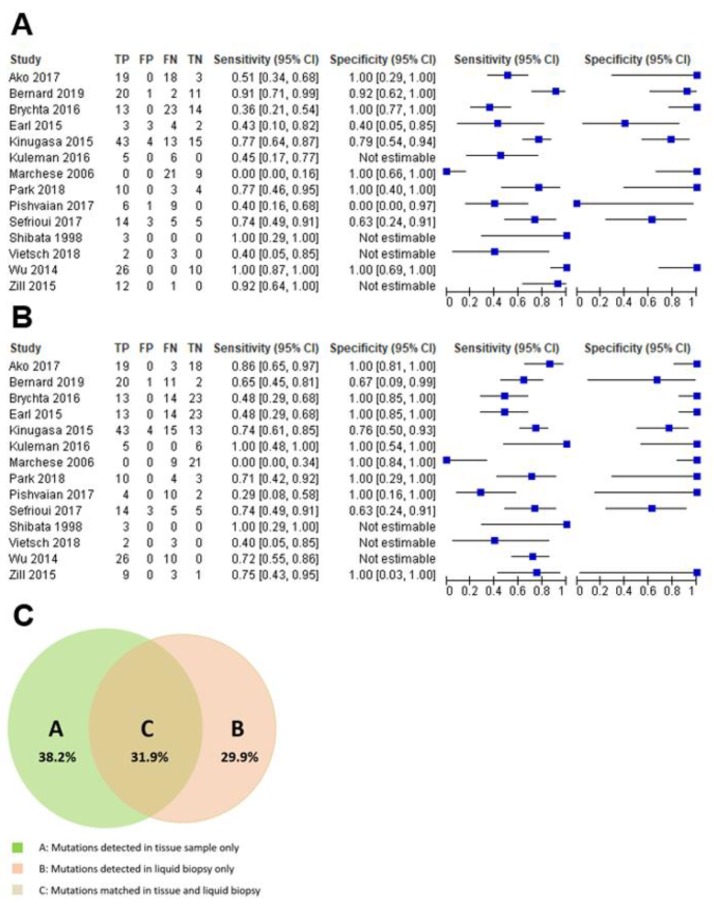
(**A**) shows the sensitivity and specificity meta-analysis plot for the appropriateness of liquid biopsy compared to tissue specimen, whereas (**B**) shows the same plot, but obtained analyzing data regarding *KRAS* mutational status only. (**C**) is a Venn diagram showing, for studies based on next-generation sequencing, the concordance rate of all mutations detected by the analysis on tissue specimens and by the analysis on liquid biopsy. The area on the left (green area) indicates mutations detected only in tissue samples, the area on the right (red area) indicates mutations detected only in liquid biopsy, whereas the middle area indicates mutations detected in both tissue samples and liquid biopsy.

As reported in Figure 1A and Figure 2A, the overall pooled sensitivity and specificity were 0.70 (95% CI, 0.63–0.76) and 0.86 (95% CI, 0.77–0.93) respectively. It has to be clear that these summarizing values are referring to sensitivity and specificity with respect to concordance between LB and tissue molecular analyses and not with respect to cancer diagnosis more generally.

**Figure 3:**
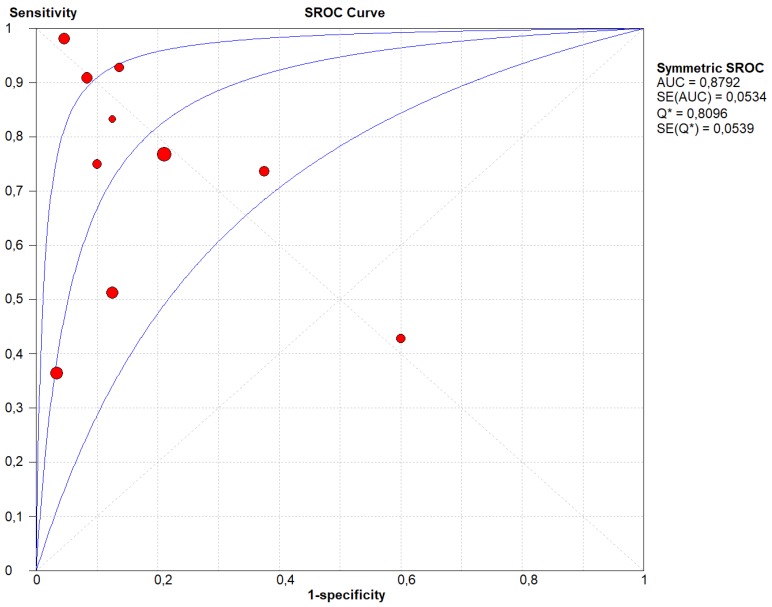
Summary Receiver Operating Curve (SROC) of the appropriateness liquid biopsy, taking tissue specimen as the outcome. In this figure, the blue lines represent the AUC (central line) with its 95% CI (external lines) calculated with a meta-analytic approach, while red dots represent the sensitivity and specificity data for each study.

Our results showed that LR+ was 3.85 (95% CI, 1.87–7.90), LR- was 0.34 (95% CI, 0.19–0.62) and DOR was 15.84 (95% CI, 4.98–50.38) (Figure 1A). Between-study heterogeneity was significant in the sensitivity, specificity, and the DOR (I-square estimated to respectively 84.2%, 54.6%, and 50.3%), but the stage of the pancreatic cancer (I–II vs. III–IV) did not explain any of these high I-squares (beta = 0.28, *p* = 0.28 for sensitivity; beta = 0.89, *p* = 0.45 for specificity; beta = 0.51, *p* = 0.28 for DOR). Similar results were evident for the type of molecular analysis of both tissue samples and LB (divided in PCR and NGS) (beta = −4.24, *p* = 0.17 for sensitivity; beta = 2.81, *p* = 0.32 for specificity; beta = −3–24, *p* = 0.68 for DOR). We did not find any evidence of threshold effect (Spearman correlation coefficient: 0.157; *p* = 0.756). Figure 3 shows the corresponding SROC curve with AUC of 0.880 indicating that LB is capable of identifying the mutations present in pancreatic cancer with a relatively high accuracy.

**Figure 4:**
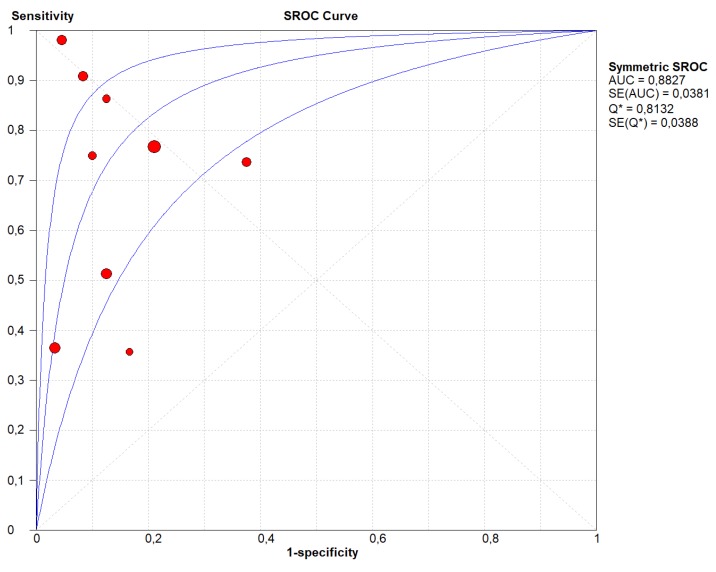
Summary Receiver Operating Curve (SROC) of the appropriateness liquid biopsy, taking *KRAS* mutational status as the outcome. In this figure, the blue lines represent the AUC (central line) with its 95% CI (external lines) calculated with a meta-analytic approach, while red dots represent the sensitivity and specificity data for each study.

Figure 1B and Figure 2B show the same analyses, taking *KRAS* mutation as the outcome. As summarized in Table S2, the TP were 169, the FP 11, TN 78 and the FN 111. The overall pooled sensitivity and specificity were 0.65 (95% CI, 0.59–0.71) and 0.91 (95% CI, 0.83–0.96) respectively. Our results showed that LR+ was 3.93 (95% CI, 2.33–6.62), LR- was 0.39 (95% CI, 0.25–0.62) and DOR was 16.41 (95% CI, 7.04–38.29) (Figure 1B). We observed a significant heterogeneity for sensitivity (I-square = 87.0%), but not for specificity and the DOR (I-square = 46.0% and 0%, respectively). Again, the stage of the cancer did not explain the heterogeneity found (beta = 0.89, *p* = 0.26), as well as the type of molecular analysis (PCR vs. NGS, beta = −2.82, *p* = 0.46). We did not find any evidence of threshold effect (Spearman correlation coefficient: 0.148; *p* = 0.622). Figure 4 shows the SROC curve of *KRAS* only, with AUC of 0.882 indicating a relatively high accuracy also for LB investigating *KRAS* only.

For NGS studies, the concordance rate of all mutations detected by multi-genes panels analyzing tissue specimens vs. LB was 31.9%, while 38.2% of mutations were detected on tissue specimens only and 29.9% on LB only (Figure 2C).

## 3. Discussion

In the present meta-analysis, 14 different studies containing a total of 369 patients, with matched molecular analysis of PDAC tissue specimens and LB, were analyzed. The overall pooled sensitivity and specificity of LB compared to the mutational analysis on tumor tissue were 0.70 (95% CI, 0.63–0.76) and 0.86 (95% CI, 0.77–0.93), respectively. Considering those studies that analyzed *KRAS* mutations only, the sensitivity was slightly lower (0.65; 95% CI, 0.59–0.71), but the specificity was higher (0.91; 95% CI, 0.83–0.96). Notably, the SROC curves indicate that, compared to tissue specimens, LB demonstrated a high accuracy in determining the mutational asset of PDAC (AUC of 0.880 for all studies and of 0.882 for those regarding *KRAS* only).

A recent study indicated that a large body of literature has been produced in recent years related to LB in cancer, but less than 1% regarded PDAC [35]. Despite this restricted number of studies on PDAC compared to other cancer types (e.g., lung and breast cancers), LB represents also for this cancer a very promising tool in the era of precision medicine. The values obtained by the quantitative synthesis of this meta-analysis, indeed, does indicate a potential role of LB in the management of PDAC patients.

The major advantage of LB is that it does not require an invasive procedure, highlighting its very promising role firstly in the management of old and/or low-performance status patients [33]. Indeed, with a reliable radiological background, the detection of a multigene PDAC-specific molecular profiling could support the diagnosis of pancreatic cancer without the need of further and hazardous analyses. Notably, in case of the use of multigene panels for molecular analysis, LB could highlight with a reasonable certainty the presence of druggable mutations, with potential important implications for targeted therapies. Thus, also based on this meta-analysis, LB should be encouraged in all cases presenting significant contraindications to invasive or mini-invasive procedures.

Notably, higher concentrations of cfDNA and/or higher numbers of CTCs have been demonstrated in case of late-stage diseases, particularly in case of metastatic or locally-advanced PDAC [20,36,37,38,39,40,41,42]. Although in our meta-analysis a sub-stratification by stage was not possible due to statistical reasons (i.e., the rate of false positive in early stages was zero for all the studies included), the use of LB in late-stage PDAC should guarantee higher sensitivity and specificity compared to those obtained in a localized disease. This advantage may also be translated into the use of LB for the follow-up of metastatic patients during therapy, which is another potentially useful application of LB for the management of PDAC patients [12,22,32,43].

Another potential advantage of LB may be represented by the possibility of overcoming the issue linked to tumor heterogeneity, potentially showing mutations not present in a restricted area of tumor tissue. Although some studies have shown that driver gene mutations in PDAC are usually maintained during clonal evolution [44,45], this aspect may be even more evident for metastatic PDAC, in which a biopsy of the primary tumor or of the metastatic tissue cannot always represent such a complex molecular landscape [46,47,48]. In our meta-analysis, four studies presented data derived from NGS [28,29,32,34]. About one third of all mutations were detected simultaneously by sequencing on tissue specimens and LB, but 38.2% and 29.9% of all mutations were detected respectively on tissue specimens and on LB only. These significant differences could be partly explained by intra-tumor heterogeneity and by clonal evolution of cancer cells, which are well-recognized important biological issues influencing tumor representativeness by tissue specimens. Notably, Vietsch and colleagues demonstrated that cancer heterogeneity within each patient was much more evident in the mutational landscape of cfDNA than in tumor tissue DNA [32]. In their paper, indeed, they demonstrated that the majority (78%) of mutations in the cfDNA were not detected in the primary tumor tissue; this finding demonstrated that a tissue section of a given tumor could fail to represent the molecular makeup of the entire cancer. About the differences in mutation detection using PDAC tissue vs. LB, the lack of standardized and well-established procedures for the molecular analysis of LB could also have partly affected the reliability of the molecular profiling. This point has been further complicated by recent evidence that has described the detection of hotspot PDAC mutations in patients without cancer, an issue that is still under investigation [25].

Regarding the slightly higher specificity, we have found for *KRAS* compared with all genes, it is important to acknowledge that the *KRAS* mutation is not highly specific for PDAC, even if matched with histology [49]. Indeed, it can occur frequently in precursor lesions as well as in cancers of other organs, such as colorectal and lung malignancies [50,51,52]. In this regard, it is important to stretch that the specificity regarding *KRAS* that has emerged in the current meta-analysis reflects the correlation existing between the primary tumor tissue and the LB, and not the overall specificity of KRAS mutational status for PDAC diagnosis.

Despite of these important limitations, LB may become a fundamental tool in the routine clinical management of PDAC patients because of its several advantages, also highlighted by the results of this meta-analysis. However, many aspects can be better addressed in the future to improve LB sensitivity, specificity, and accuracy, allowing it to be introduced as a new and decisive tool for the development of precision medicine in the clinical care of PDAC patients [53]. As the American Society of Clinical Oncology and the College of American Pathologists have highlighted in a recommendation paper, indeed, further studies with standardized technologies are needed to introduce LB in clinical practice [54]. Of note, the introduction of the analysis with LB of epigenetic markers [55,56], of exosome-derived DNA [22,36], and of gene expression profiling/circulating proteins [35,57,58] may represent important steps in improving this kind of non-invasive approach for the molecular characterization of PDAC. Particularly, about epigenetic alterations, DNA methylation may represent a very promising horizon in this changing scenario. Recent studies had indeed shown that DNA methylation signatures were highly consistent between cfDNA and the genomic DNA from its tissue origins also in cancer models [59,60]. Furthermore, if in terms of somatic mutations a high inter-tumor heterogeneity may be found, in the same tumor category the epigenetic profiles are quite similar, and can be of great help, for example, in identifying the actual tumor origin also with LB and in metastatic settings [60]. Gai and colleagues suggested that tissue-specific methylation biomarkers are reasonably consistent across cancer patients for plasma DNA-based cancer testing [60]. Notably, Chan and colleagues reported that, using whole genome bisulfite sequencing of plasma cfDNA, the methylation profile could serve as a general approach for the diagnosis of multiple types of cancers [61]. It is also of importance to report that, besides DNA methylation, recent studies have also highlighted the promising potential of cfDNA fragmentation patterns as an emerging research direction in cancer liquid biopsy [62]. Indeed, cfDNA molecules were not randomly fragmented, but they showed strong size patterns that are associated with the nucleosome footprint, indicating that cfDNA fragmentation patterns may have certain biological and/or clinical potential in liquid biopsy. However, current knowledge on cfDNA fragmentation patterns, either from a biological basis or clinical utilities, is considerably still preliminary and without direct implications for clinical practice [62]. Another important aspect, which should be further elucidated and validated by future research, is the enhanced detection of cfDNA by specific DNA fragment size analysis (90–150 base-pairs), recently uncovered by Mouliere and colleagues [63]. Indeed, with the analysis of size-selected cfDNA, they identified clinically actionable mutations and copy number alterations that were otherwise not detected.

The limitations of our study are the overall small sample size, but this is predominantly owing to the recent development of the application of LB in PDAC studies, and the different molecular analysis that we have quantitatively summarized. Finally, the heterogeneity of our outcomes can be considered as another limitation of our study. This high heterogeneity and the differences that have emerged in both sensitivity and specificity may derive from the varying technologies applied in the studies, as well as in the different timings of LB analysis or also in differences of sequencing depths in NGS studies. Although in our meta-regression analysis we did not find significant moderators to explain this aspect, this point suggests that further studies with well-standardized techniques are needed before the introduction of LB in routine diagnostic activity.

## 4. Materials and Methods 

This systematic review adhered to the MOOSE guidelines [64] and PRISMA statement [65], following a predetermined but unpublished protocol.

### 4.1. Inclusion and Exclusion Criteria

Studies were eligible if they met the following criteria: (1) A prospective cohort or retrospective study design; (2) contained a clear comparison of mutational analysis using cancer tissue samples vs. LB in patients with pancreatic cancer; (3) contained histological or cytological diagnosis of pancreatic cancer (pancreatic ductal adenocarcinoma); and (4) were published in a peer review journal or published abstract.

Exclusion criteria were: (1) Comparison of patients with pancreatic cancer with patients without cancer (healthy volunteers, chronic pancreatitis); (2) no presence of invasive cancer (e.g., intraductal papillary mucinous neoplasm, so called IPMN); (3) no data regarding molecular analysis of tissue samples and LB in the title/abstract; (4) no data on mutational profiling; (5) diagnosis of non-epithelial malignancies (i.e., lymphomas) or of other histology other than PDAC (e.g., acinar cell carcinoma), and (6) case reports, in vitro or animal studies. We considered articles in any language. 

### 4.2. Data Sources and Literature Search Strategy

Two investigators (C.L., N.V.) independently searched PubMed, SCOPUS, and Embase until 31 January 2019. The search terms used in PubMed included combinations of the following keywords: (“pancreatic” OR “pancreas” OR “pancrea*”) AND (“cell free DNA” OR “cfDNA” OR “circulating DNA” OR “circulating tumor cells” OR “circulating tumour cell” OR “CTC” OR “CTCs”) AND (“diagnosis” OR “sensitivity” OR “specificity” OR “accuracy”). A similar search was carried out in SCOPUS and Embase. We considered the reference lists of all included articles and of previous related reviews.

### 4.3. Study Selection

Following the searches as outlined above, after removal of duplicates, two independent reviewers (C.L., N.V.) screened titles and abstracts of all potentially eligible articles. The two authors applied the eligibility criteria, considered the full texts, and a final list of included articles was reached through consensus with a third author (A.N.).

### 4.4. Data Extraction

Two authors were involved in data extraction in a standardized Microsoft Excel database. Specifically, one author (C.L.) extracted data from the included articles and a second independent author (N.V.) validated the data. For each article, we extracted information about authors, year of publication, number of patients, tumor stage, type of tissue samples, type of LB, type of molecular analysis of both tissue samples and LB (divided in PCR and NGS), number of true positive, true negative, false positive and false negative results, using the molecular analysis on tissue samples as reference.

### 4.5. Outcomes

The primary outcomes were sensitivity, specificity, positive and negative likelihood ratios, diagnostic odds ratio (DOR), and the area under the curve (AUC) of the molecular analysis with LB. For those studies which used multigene-panels of next-generation sequencing for molecular analysis, the concordance rate between the molecular profiles of tissue samples and LB was also reported. A similar (secondary) analysis of concordance was also performed considering the mutational status of the *KRAS* gene only.

### 4.6. Assessment of Study Quality

Based on the revised quality assessment of diagnosis, accuracy studies-2 (QUADAS-2) criteria, the included articles were evaluated as at high risk (−) or low risk (+) by four key domains: Patient selection, index test, reference standard, and flow and timing [66].

### 4.7. Data Synthesis and Statistical Analysis

We used RevMan Manager 5.3 and Meta-Disc software 5.1.32 to conduct this meta-analysis. All the studies reported the data regarding true positive (TP), true negative (TN), false positive (FP) and false negative (FN). Therefore, we were able to calculate the pooled sensitivity (TP/TP + FN), specificity (SPE) (TN/TN + FP), negative likelihood ratio (LR−), positive likelihood ratios (LR+) and DOR with their 95% confidence intervals. At the same time, we constructed the summary receiver operator characteristic (SROC) curve and calculated the area under the SROC curve based on the sensitivity and specificity of each study. In case of high heterogeneity (as indicated by an I^2^ > 50%), we performed a meta-regression analysis, taking the stage of the tumor (I–II vs. III–IV) and the type of molecular analysis of both tissue samples and LB (divided in PCR and NGs) as potential moderators.

For those studies that used multi-genes panel for DNA sequencing, we also compared the concordance rate of mutations detected by the analysis on tissue specimens and the analysis on LB, designed a summarizing Venn diagram.

## 5. Conclusions

To conclude, although further studies are needed to improve the standardization and the applicability of LB, we have demonstrated in this meta-analysis that this non-invasive approach can serve as surrogate to tissue in the molecular profiling of PDAC, because of its relatively high sensitivity, specificity, and accuracy. Thus, LB could be introduced in clinical practice and should be further explored towards the development of new precision medicine approaches against pancreatic cancer.

## Figures and Tables

**Table 1:** Summarizing table of the most relevant features of the studies included in this meta-analysis.

First Author of the Study, Year [21,22,23,24,25,26,27,28,29,30,31,32,33,34]	N. of Patients	Stage	Type of Tissue Specimen	Molecular Test for Tissue Specimen and Genes	Time Point of Tissue and Liquid Biopsy Test and Genes	Type of Liquid Biopsy	Molecular Test for Liquid Biopsy	TP	FP	TN	FN
Ako, 2017 [21]	40	I–II: 60%, III–IV: 40%	16 SR and 24 EUS-FNA	PCR, KRAS	The same time	Plasma and serum, cfDNA	Droplet PCR, KRAS	19	0	3	18
Bernard, 2019^a^ [22]	34	I–II: 68%, III–IV: 32%	22 SR and 12 EUS-FNA	PCR, KRAS	The same time	Blood for cfDNA	Droplet digital PCR, KRAS	20	1	11	2
Brychta, 2016 [23]	50	I–II: 82%, III–IV: 18%	SR	Chip-based digital PCR, KRAS	The same time	Plasma (cfDNA)	Chip-based digital PCR, KRAS	13	0	14	23
Earl, 2015 [24]	12	NA	SR	PCR, KRAS	The same time	Plasma (cfDNA)	Droplet digital PCR, KRAS	3	3	2	4
Kinugasa, 2015 [25]	75	I–II: 3%, III–IV: 97%	EUS-FNA	PCR, KRAS	The same time	Serum (cfDNA)	Droplet digital PCR, KRAS	43	4	15	13
Kulemann, 2016 [26]	11	I–II: 91%, III–IV: 9%	NS	PCR, KRAS	Retrospective	Blood with isolation and analysis of CTCs	PCR, KRAS	5	0	0	6
Marchese, 2006 [27]	30	I–II: 83%,III–IV: 17%	25 SR, 5 EUS-FNA	rflp-PCR KRAS	The same time	Serum (cfDNA)	rflp-PCR KRAS	0	0	9	21
Park, 2018^a^ [28]	17	I–II: 18%,III–IV: 82%	EUS-FNA	PCR, KRAS	The same time	Plasma (cfDNA)	PCR, KRAS	10	0	4	3
Pishvaian, 2017^a,^* [29]	16	I–II: 0%,III–IV: 100%	EUS-FNA of pancreas or metastasis	321 genes panel NGS	During treatment	cfDNA	68 genes panel NGS	6	1	0	9
Sefrioui, 2017 [30]	27	NS	EUS-FNA/biopsy/SR	Digital PCR, KRAS	The same time	Plasma (cfDNA)	Digital PCR, KRAS	14	3	5	5
Shibata, 1998 [31]	3	I–II: 66.6%, III–IV: 33.3%	NS	nPCR, KRAS	The same time	Peripheral blood (CTCs separation)	nPCR, KRAS	3	0	0	0
Vietsch, 2018^a,^* [32]	5	I–II: 100%, III–IV: 0%	SR	56 genes panel NGS	LB before surgery	cfDNA	56 genes panel NGS	0	0	0	5
Wu, 2014 [33]	36	NS	NS	COLD-PCR, KRAS	The same time	Plasma (cfDNA)	COLD-PCR, KRAS	26	0	10	0
Zill, 2015 [34]	13	NS	EUS-FNA	NGS	The same time	Plasma (cfDNA)	54 genes panel NGS	12	0	0	1
**Total**	**369**	**I–II: 57%, III–IV: 43%**			**11 studies: same time, 3 studies: other times**	**12 studies: cfDNA from blood, 2 studies: CTCs separation**		**174**	**12**	**73**	**110**

**Abbreviation**: TP: true positive, FP: false positive, TN: true negative, FN: false negative; SR: surgical resected specimen; EUS-FNA: endoscopic ultrasound-guided fine-needle aspiration; cfDNA: circulating-free DNA; CTCs: circulating tumor cells; NS: not specified; rflp-PCR: restriction fragment length polymorphism-polymerase chain reaction; NGS: targeted next-generation sequencing for specific pancreatic cancer genes; nPCR: nested PCR; LB: liquid biopsy; COLD-PCR: co-amplification-at-lower-denaturation-temperature polymerase chain reaction; **Note**: Bernard, 2019^a^ refers to the analysis of exoDNA in liquid biopsy; Park, 2018^a^ refers to the analysis using PCR; Pishvaian, 2017^a^ refers to a cohort of patients in which cfDNA has been analyzed; Vietsch, 2018^a^ refers to patients whose liquid biopsy has been analyzed before surgical resection of pancreatic tumor; * in these studies, the rate of concordance of mutations between tumor tissue and liquid biopsy has been shown taking into account the four most important genes in pancreatic cancer (*KRAS*, *TP53*, *SMAD4*, and *CDKN2A*).

## Supplementary Materials

The following are available online at https://www.mdpi.com/2072-6694/11/8/1152/s1, Figure S1: PRISMA checklist for this meta-analysis, Table S1: QUADAS-2 tool: Risk of bias and applicability judgments of the present meta-analysis, Table S2: Data considering the mutational status of *KRAS* gene only.
