# Supplementary material for: Liquid Biopsy as Surrogate for Tissue for Molecular Profiling in Pancreatic Cancer: A Meta-Analysis Towards Precision Medicine
Source: Cancers (Basel). 2019 Aug 10;11(8):1152. doi: 10.3390/cancers11081152 (PMC6721631; doi:10.3390/cancers11081152)
Supplement: Supplementary file 1 [file cancers-11-01152-s001.pdf]

Supplementary Materials

Liquid Biopsy as Surrogate for Tissue for Molecular Profiling in Pancreatic Cancer: A Meta-Analysis Towards Precision Medicine

Claudio Luchini, Nicola Veronese, Alessia Nottegar, Vera Cappelletti, Maria G. Daidone, Lee Smith, Christopher Parris, Lodewijk A. A. Brosens, Maria G. Caruso, Liang Cheng, Christopher L. Wolfgang, Laura D. Wood, Michele Milella, Roberto Salvia and Aldo Scarpa

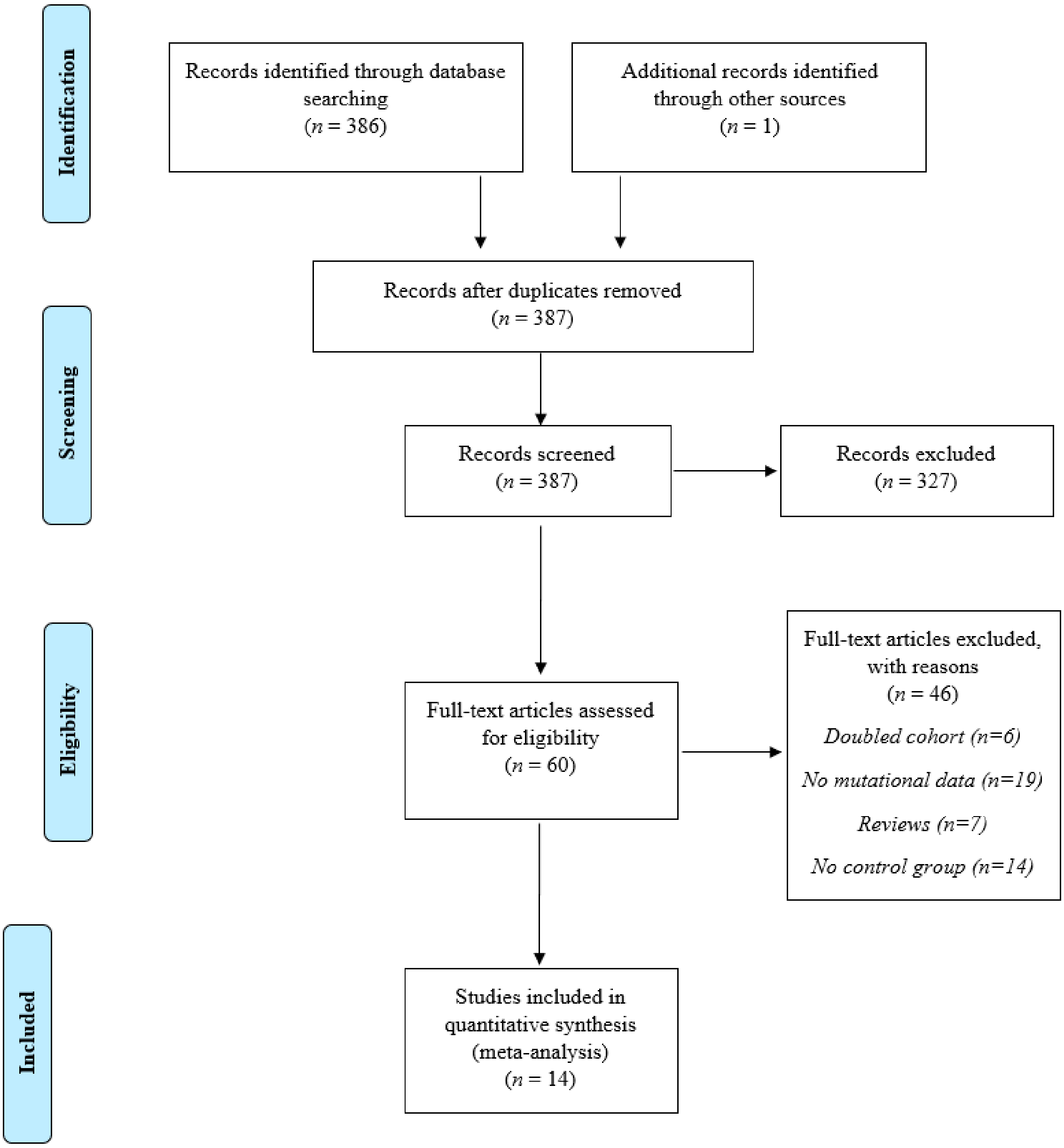

Figure S1. PRISMA checklist for this meta-analysis.

Table S1. QUADAS-2 tool: Risk of bias and applicability judgments of the present meta-analysis.

Study	Risk of Bias				Applicability Judgments		
	**Patients Selection**	**Index Test**	**Reference Standard**	**Flow and Timing**	**Patients Selection**	**Index Test**	**Reference Standard**
Ako, 2017	+	+	+	+	+	+	+
Bernard, 2019	+	+	+	+	+	+	+
Brychta, 2016	+	+	+	+	+	+	+
Earl, 2015	+	+	+	+	+	+	+
Kinugasa, 2015	+	+	+	+	+	+	+
Kulemann, 2016	+	+	+	-	+	+	+
Marchese, 2006	+	+	+	+	+	+	+
Park, 2018	+	+	+	+	+	+	+
Pishvaian, 2017	+	+	+	-	+	+	+
Sefrioui, 2017	+	+	+	+	+	+	+
Shibata, 1998	+	+	+	+	+	+	+
Vietsch, 2018	+	+	+	-	+	+	+
Wu, 2014	+	+	+	+	+	+	+
Zill, 2015	+	+	+	+	+	+	+

Table S2. Data considering the mutational status of *KRAS* gene only.

First Author of the Study, Year	N. of Patients	TP	FP	TN	FN
Ako, 2017	40	19	0	3	18
Bernard, 2019	34	20	1	11	2
Brychta, 2016	50	13	0	14	23
Earl, 2015	12	3	3	2	4
Kinugasa, 2015	75	43	4	15	13
Kulemann, 2016	11	5	0	0	6
Marchese, 2006	30	0	0	9	21
Park, 2018[a]	17	10	0	4	3
Pishvaian, 2017[a*]	16	4	0	2	10
Sefrioui, 2017	27	14	3	5	5
Shibata, 1998	3	3	0	0	0
Vietsch, 2018[a*]	5	0	0	0	5
Wu, 2014	36	26	0	10	0
Zill, 2015	13	9	0	3	1
**Total**	**369**	**169**	**11**	**78**	**111**

© 2019 by the authors. Licensee MDPI, Basel, Switzerland. This article is an open access article distributed under the terms and conditions of the Creative Commons Attribution (CC BY) license (http://creativecommons.org/licenses/by/4.0/).
